# Enzymatic Activity of Soil on the Occurrence of the Endangered Beetle *Cheilotoma musciformis* (Coleoptera: Chrysomelidae) in Xerothermic Grasslands

**DOI:** 10.3390/insects15050307

**Published:** 2024-04-25

**Authors:** Barbara Futa, Mariusz Kulik, Łukasz Kajtoch, Miłosz A. Mazur, Małgorzata Jaźwa, Radosław Ścibior, Justyna Wielgos

**Affiliations:** 1 Institute of Soil Science, Environment Engineering and Management, University of Life Sciences in Lublin, Leszczyńskiego 7, 20-069 Lublin, Poland; barbara.futa@up.lublin.pl; 2 Department of Grassland and Landscape Planning, University of Life Sciences in Lublin, Akademicka 15, 20-950 Lublin, Poland; mariusz.kulik@up.lublin.pl (M.K.); justynawielgos.up@gmail.com (J.W.); 3 Institute of Systematics and Evolution of Animals, Polish Academy of Sciences, Sławkowska 17, 31-016 Cracow, Poland; lukasz.kajtoch@gmail.com; 4 Institute of Biology, University of Opole, Oleska 22, 45-050 Opole, Poland; milosz@uni.opole.pl (M.A.M.); malgorzata.jazwa@uni.opole.pl (M.J.); 5 Department of Zoology and Animal Ecology, University of Life Sciences in Lublin, Akademicka 13, 20-950 Lublin, Poland

**Keywords:** dehydrogenases, urease, acid phosphatase, alkaline phosphatase, total organic carbon content, total nitrogen content, leaf beetle

## Abstract

**Simple Summary:**

This study aimed to elucidate the factors contributing to the limited presence of *Cheilotoma musciformis* in Poland, with a specific focus on soil characteristics affecting both vegetation and insect populations. It examined how soil enzyme activity influences the occurrence of *Ch. musciformis* in the xerothermic grasslands of Southern Poland. Sites inhabited by the beetle were typically subject to extensive grazing by farm animals or recent bush clearance, contrasting with control plots situated on unused or overgrazed xerothermic grasslands. Soils in beetle-inhabited sites exhibited significantly higher levels of enzyme activity, total organic carbon, and total nitrogen, along with lower pHKCl compared to control sites. These findings suggest the beetle’s reliance on extensively grazed xerothermic grasslands. Given that grazing practices influence the behavior of preferred host plant species, effective protection planning for *Ch. musciformis*-inhabited grasslands should carefully consider changes in soil biochemical properties and vegetation structure.

**Abstract:**

This work attempts to find the reasons for the rather limited range of occurrence of *Cheilotoma musciformis* in Poland, based on soil properties, which affects both the plant cover and the entomofauna. The aim of the study was to assess the influence of soil enzyme activity on the occurrence of *Ch. musciformis* in xerothermic grasslands in Southern Poland. The sites inhabited by the beetle were most often extensively grazed by farm animals or had recently been cleared of bushes. The control plots were in wasteland. The soils of most sites with *Ch. musciformis* were characterized by significantly higher activity of the tested enzymes and higher content of total organic carbon and total nitrogen, as well as lower pHKCl compared to the control sites. The higher enzymatic activity of soils in sites with the beetle than in the control sites may indicate the dependence of the occurrence of this beetle on the presence of patches of extensively grazed xerothermic grasslands. Grazing influences the behavior of preferred host plant species. Therefore, when planning active protection of xerothermic grasslands inhabited by *Ch. musciformis*, changes in the biochemical properties of the soil and vegetation structure should be taken into account.

## 1. Introduction

The genus *Cheilotoma* (Chevrolat, 1837) is represented in the Palearctic by six species [1], of which only *Ch. erythrostoma* (Faldermann, 1837) and *Ch. musciformis* (Goeze, 1777) occur in Europe [2,3,4]. *Ch. musciformis* is more widely distributed and reaches its northernmost range in Poland [2,5]. The distribution range of the nominative subspecies can be considered south-Euro-Siberian, as it covers the area from France, through most of the countries of Southern Europe, the Levant, Asia Minor, and Central Asia to Mongolia [1,6], while north of the Carpathians and Bohemian Massif there are only three known, geographically isolated and probably strongly disappearing populations—German, Polish, and Western Ukrainian, located 300–500 km apart [7]. These isolated localities of *Ch. musciformis* are currently up to several hundred kilometers outside the main range of occurrence of the species [8]. This is related to the extrazonal distribution of steppe formations in Europe [9]. Information about the occurrence of this species in Poland has been known since the end of the 19th century from Kraków–Wieluń Upland (Ojców region) and from the 20th century to the present also from the Nida Basin (the Małopolska Upland) [10]. In the years 2009–2014, this taxon was intensively searched for its known area of occurrence, and was found in a total of 31 sites, including two new ones [7]. According to Warchałowski [2], in Poland this beetle was caught on *Onobrychis viciifolia*, exclusively in xerothermic grasslands. In other parts of the range, *Rumex* spp. is also mentioned [11,12]. Recent genetic studies [13,14] showed that this species in Poland feeds primarily on *Onobrychis* spp., and occasionally also on other legumes. Most reports of the presence of *Ch. musciformis* in the sites currently studied concerned its catches and observations on *Onobrychis viciifolia*, which, as a kenophyte, inhabits the slopes and limestone outcrops of Central Poland [15]. Recently, this plant went from cultivation to grasslands, so it could not have been the original host plant of the beetle in our country. This is because genetic data indicate a much longer history of isolation of the Polish population of the beetle, perhaps dating back to the last ice age [8]. In the studied area, *Onobrychis viciifolia* has probably recently replaced another host plant of this genus—*Onobrychis arenaria*. It is a native, but currently rare and endangered element of the xerothermic grasslands [16]. Mazur et al. [7] pointed out that for the conservation status of this species, it will be very important to know other (still unexplored) ecological or ethological factors limiting its distribution, especially since the beetle has never been found on *O. viciifolia* outside of the xerothermic habitats of the studied area (even equally well preserved in neighboring macroregions). In the past, this plant was common and was sown as fodder. Perhaps the occurrence of the beetle only in xerothermic grasslands should be associated with the thermal, topographic and edaphic conditions of turfs, as was reported in the case of other grassland beetles [17,18]. It is also likely that chemical composition of the host plant, which varies depending on the substrate [19], affects the selection of the habitat, particularly by the larvae. According to Erber [20], most Cryptocephalinae larvae feed on the ground, feeding on leaf debris or rotting plant material, and in the Clytrini tribe (to which the genus *Cheilotoma* belongs), some genera are partially or completely myrmecophilous, or their preimaginal stages live in the litter. *Ch. musciformis* larvae occur in steppe habitats under stones and among rock debris [21]. It is not yet known at what stage this taxon hibernates, but the appearance of the egg and larva is known [21,22].

Today, steppe habitats in Central and Western Europe are the extrazonal remains of refugia of warm steppes, which were widely distributed in this area during the last glaciations [9,23,24]. In Poland, they are home to many rare and endemic thermophilic species. Mitochondrial and nuclear markers indicate that the Polish population of this taxa is genetically different from the closest populations of Ukraine and Slovakia [7,8]. At the same time, it shows very low genetic diversity, which could indicate the existence of a separate evolutionary unit of this beetle, perhaps at the level of a subspecies [7].

It seems that habitat isolation and genetic factors pose a significant threat to the national population [8]. *Ch. musciformis* is a rare species not only in Poland, which was mentioned in several studies on endangered species: in the Red List of Endangered and Endangered Animals [25], or in the Polish Red Data Book of Animals—Invertebrates with the category—endangered (EN) [26]. It has had the status of “2” (=highly threatened) in Germany for many years [27,28,29]. In the Czech Republic, it is a critically endangered species (CR) [30]. The Polish population of *Ch. musciformis* is in decline now and its range has shrunk by approximately 30% over the last dozen or so years (authors’ own data). The species has disappeared from the Kraków–Częstochowa Upland and from most locations in the Kielce Upland, currently located the northernmost in the country, with the exception of Góra Rzepka [7,31], where this beetle was found in 2023 [Bidas, M., personal information], after two decades of missing records (probably as a result of effective conservation measures—clearing the slope of bushes). The species can actively disperse because it is a flight beetle, although it flies very reluctantly and rarely (authors’ own observations). Despite this, its range in Poland has not increased for nearly 200 years, which is confirmed by long-term entomological collections from this region, deposited in the Natural History Museum of the Institute of Systematics and Evolution of Animals Polish Academy of Sciences in Krakow.

For several previous years, additional research was carried out in the analyzed area of the Małopolska Upland to verify the localities of *Ch. musciformis*. The species was found at several sites, including several newly discovered ones, where no searches had been carried out before. The research included sites with similar habitat conditions, where calcareous xerothermic grasslands of the *Festuco-Brometea* class were found, which are one of the most endangered habitats of the Natura 2000 network in Europe, being a refuge for many rare and endangered species of flora and fauna [32]. The appropriate form of protection for xerothermic grasslands is to conduct extensive grazing with a small number of livestock [33] and to leave fragments of grasslands without grazing in subsequent years, which ensures the development of vegetation throughout the season. Extensive grazing affects not only plant cover, increasing floristic diversity [34], but also the physicochemical and biochemical properties of soils [35,36]. Soil quality and fertility play an important role in habitat development [36]. The physical, chemical, and biological properties of the soil determine its fertility. These include grain size, moisture, carbon and nitrogen content, micro- and macroelement content, pH (acidity or basicity), salinity, structure, and number of soil organisms and enzymatic activity [37,38]. Analysis of these parameters allows for the observation of changes occurring in the soil environment. Physicochemical properties alone are not sensitive enough to track relatively subtle changes in soil quality, as these parameters usually change very slowly [39]. Therefore, in soil assessment, in addition to physicochemical parameters, the following are used: soil enzymes that participate in the flow of energy and nutrient circulation in the environment, i.e., C, N, S, and P. In addition, soil enzymes respond quickly to small changes in soil conditions and provide information about subtle changes in soil quality and fertility [40,41]. Tests based on soil enzyme activity are considered useful bioindicators of soil health due to their close relationship to soil properties and ease of measurement [42].

For this reason, an attempt was made to find the reasons for the rather limited range of occurrence of *Ch. musciformis* in Poland, starting with the soil, as a key element of the habitat that affects both the plant cover and the entomofauna. The purpose of the study was to assess the influence of soil enzymatic activity on the occurrence of *Ch. musciformis* in xerothermic grasslands in Southern Poland. The study assessed the activity of dehydrogenases (DhA), urease (UrA), acid phosphatase (PhacA) and alkaline phosphatase (PhalA), as well as the content of organic carbon (TOC) and total nitrogen (TN) and pH in 1 moldm^−3^ KCl (pH_KCl_).

## 2. Materials and Methods

### 2.1. Study Area

**Figure 1:**
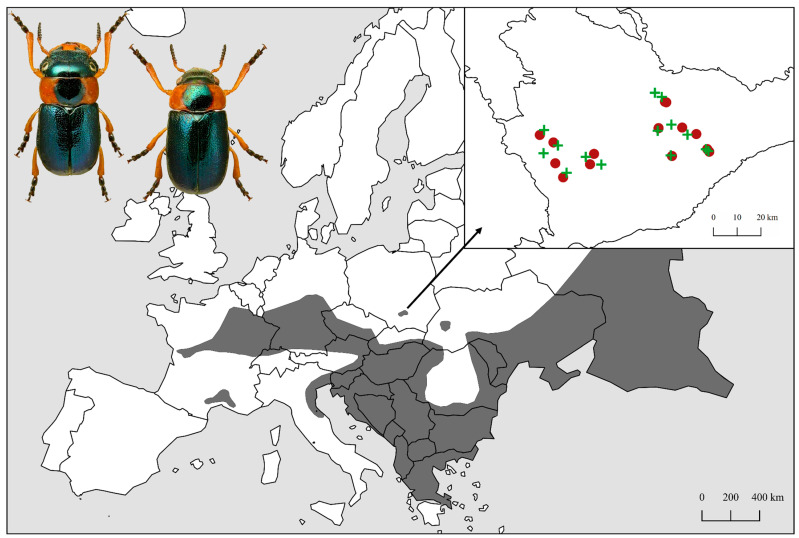
Distribution of *Cheilotoma musciformis* (**left**: male, **right**: female) in Europe (gray) and study sites in Poland (the Nida Basin macroregion—green plus signs—beetle sites [M], red dots—control sites [C]). Beetle photograph taken by Prof. Lech Borowiec—Iconografia Coleopterorum Poloniae (http://cassidae.uni.wroc.pl/Colpolon/Foto/Cheilotoma%20musciformis.jpg) (accessed on 6 March 2024). Illustration based on the free and open source QGIS 2.18 software (https://www.qgis.org/en/site/); Europe background map source: Cosmographics Ltd., United Kingdom (http://cosmographics.co.uk).

In Poland, *Cheilotoma musciformis* occurs almost exclusively in the Nida Basin: on the Pińczów Hummock (3 sites), in the Solec Basin (6), on the Wodzisław Hummock (2), on the Proszowice Plateau (1) and on the Miechów Upland (8). The only site on the Kielce Upland has been preserved in the Świętokrzyskie Mts. (Figure 1 and Supplementary Materials KMZ file).

### 2.2. Beetle Sampling

**Figure 2:**
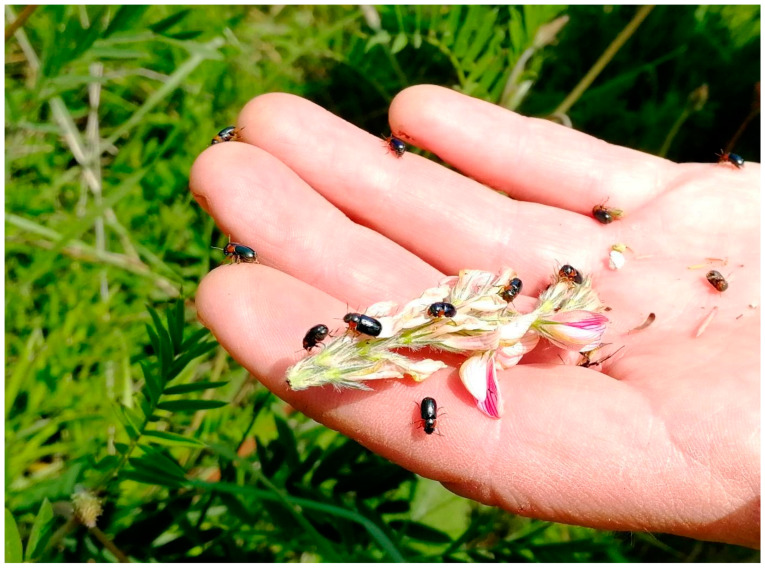
Imagines of *Cheilotoma musciformis*.

*Beetles* (Figure 2) were collected along several hundred-meter transects throughout the grassland using a sweep net, which is a standard method for collecting beetles living on herbaceous vegetation. Three inspections were carried out on each grassland on 21–29 May and 20–23 June 2022, and repeated on 26–28 May 2023. During each inspection, 600 sweeps of the sweep net were made in patches of xerothermic vegetation, with particular emphasis on sampling among legumes (mainly *Onobrychis* spp.). Beetles were selected from the sweep net using a hand-held suction device, identified as to the species, and immediately released into the environment at the collection site.

### 2.3. Field Study

**Figure 3:**
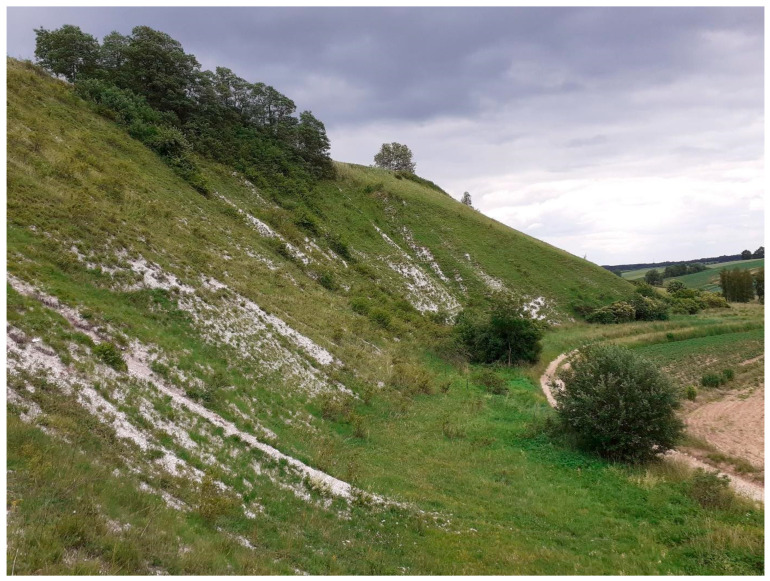
Sample of xerothermic grassland with occurrence of *Cheilotoma musciformis* (M).

**Figure 4:**
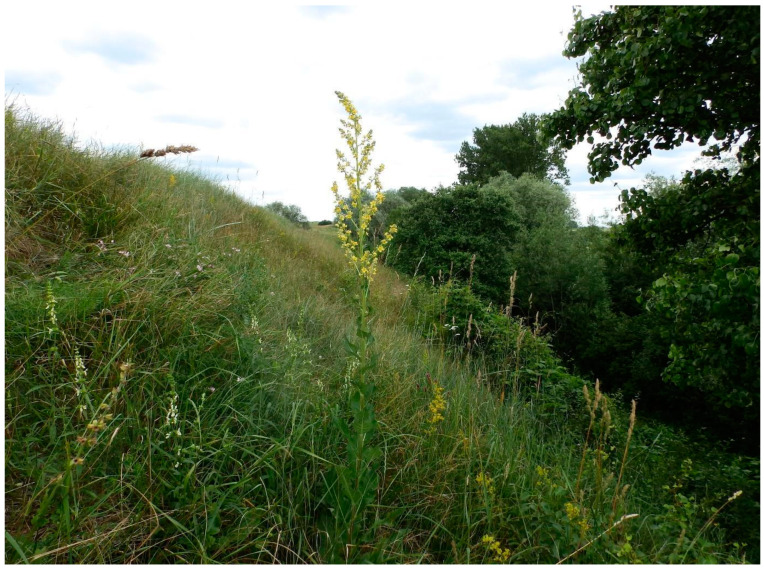
Sample of xerothermic grassland without occurrence of *Cheilotoma musciformis* (C).

The research area was soils in the xerothermic grassland habitat (located in Świętokrzyskie and Lesser Poland Voivodeships in Southern Poland) at selected sites (Figure 1 and Supplementary Materials KMZ file) (N = 14) where *Ch. musciformis* (M) was found (Figure 3), and in grasslands where this beetle was not found (N = 14) (C)—control (Figure 4). Due to their unique natural values, these areas have been covered by various forms of nature protection, including Natura 2000 areas or nature reserves. The areas were diversified terrain (mostly steep slopes), with calcaric cambisols, rendzic leptosols, and calcaric leptosol [43]. Soil material for laboratory tests was collected on 26–28 May 2023 from the 0–20 cm layer, during stable weather conditions. Five soil samples were taken from each study site and mixed to give a single sample for analysis from each site. When collecting, preparing, and storing soil material, the principles set out in the ISO 18400 [44] standard were followed. Each sample was tested in three replications.

### 2.4. Laboratory Tests

The work examined the activity of four enzymes that catalyze the decomposition of compounds rich in carbon, nitrogen, and phosphorus. Dehydrogenases (DhA; EC 1.1) participate in the biogeochemical carbon cycle in the environment and urease (UrA; EC 3.5.1.5) participates in the nitrogen metabolism cycle. However, acid (APhac; EC 3.1.3.2) and alkaline phosphatases and (APhal; EC 3.1.3.1) are involved in the decomposition of phosphorus compounds. The activity of dehydrogenases (DhA) was determined by the Thalmann method [45] using a 1% solution of TTC (2,3,5-triphenyl tetrazolium chloride) as a substrate. Acid and alkaline phosphatases (PhacA and PhalA) were determined according to Tabatabai and Bremner [46] using a 0.8% solution of PNPP (disodium p-nitrophenyl phosphate) as a substrate in buffer pH 6.5. UrA (EC 3.5.1.5) was determined after Zantua and Bremner [47] using a 2.5% urea solution as a substrate. Enzyme activities were determined calorimetrically using a CECIL CE 2011 spectrophotometer (Cecil Instruments, Cambridge, UK). Activity values are given in terms of dry weight of soil dried at a temperature of 105 °C.

Chemical analyses consisted of determining the following parameters: pH, the content of total organic carbon (TOC) and total nitrogen (TN). The pH value of the soil was determined at 1 moldm^−3^ KCl using a pH meter (ELMETRON, Zabrze, Poland) [48]. The content of TOC was investigated in the TOC-VCSH apparatus with an SSM-5000A module (Shimadzu Corp., Kyoto, Japan) [49]. The TN content was determined by the modified Kjeldahl method. For this purpose, a Kjeltech TM 8100 distillation unit [50] was used.

### 2.5. Data Analysis

Statistical analysis of the study results was performed using Microsoft Office Excel 2019 and Statistica PL 13.3 (TIBCO Software Inc., Palo Alto, CA, USA). Arithmetic means and standard deviation (SD) for individual variants were calculated. Statistical assessment of the variability of the results was performed using one-way analysis of variance (ANOVA). The significance of differences between mean values was verified on the basis of the *t*-Tukey test. Box plots were created to analyze the spatial distribution of soil properties. For the parameters studied, the Pearson linear correlation coefficient value (r) was calculated, with a significance level of *p* < 0.05. Spearman’s correlation coefficient between soil chemical parameters and soil enzyme activity was also calculated, and the obtained results were presented graphically. Additionally, the principal component analysis (PCA) was adopted to visualize the relationship between the biochemical variables of the soil examined and the presence of leaf beetles. Finally, Generalized Linear Models (GLM) were built to assess the impact of chemical components (only those with a correlation coefficient below 0.6, for the remaining PC1 models were used). The binomial distribution was used to determine the presence/absence of leaf beetles, and the Akaike Information Criterion (AIC) was adopted to rank the models.

## 3. Results

### 3.1. pH_KCl_ Values of the Tested Soils

**Figure 5:**
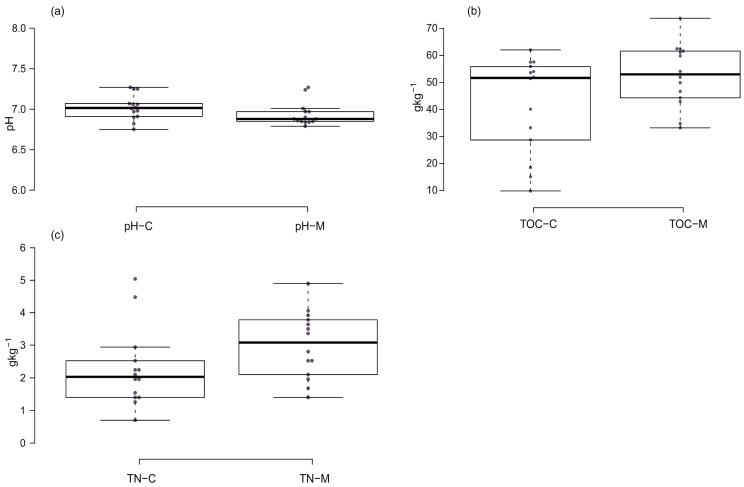
Box plots of soil chemical properties: (**a**) pH in 1 moldm^−3^ KCl (pH_KCl_), (**b**) content of total organic carbon (TOC), and (**c**) content of total nitrogen (TN). Explanations: M—sites occupied by *Cheilotoma musciformis*, C—control (unoccupied) sites. Solid centre line—the medians; box limits—the 25th and 75th percentiles; whiskers—1.5 times the interquartile range from the 25th and 75th percentiles, dots—outliers.

The test soils were characterized by a neutral and slightly alkaline reaction. The average pH_KCl_ values of surface soils with *Ch. musciformis* (M) ranged from 6.79 to 7.27 (average 6.94), and the pH_KCl_ of surface soils without the beetle (C) ranged from 6.75 to 7.27 (average 7.02) (Figure 5 and Table 1). Statistically significantly higher pH_KCl_ values were found in the soils of most sites C than in the soils of sites (M). In the case of five test sites, opposite results were obtained. The differences in exchangeable acidity of soils found in individual research areas could be related, among others, to their different grain size and properties of the parent rock or the way they are used. However, when analyzing the mean values for the entire sample, no significant differences were observed between sites M and C.

### 3.2. Content of Total Organic Carbon (TOC) and Total Nitrogen (TN) in Tested Soils

The amounts of TOC and TN in the soils of the *Ch.musciformis* (M) area ranged, respectively, 33.20–73.78 gTOCkg^−1^ (average 52.81 gTOCkg^−1^) and 1.68–4.90 gTNkg^−1^ (average 3.09 gTNkg^−1^). However, the soils of the control sites contained from 15.08 to 62.06 gTOCkg^−1^ (average 42.83 gTOCkg^−1^) and from 0.70 to 5.04 gTNkg^−1^ (average 2.19 gTNkg^−1^) (Figure 2, Table 1). In most cases, the soils of M sites were characterized by statistically significantly higher TOC and TN contents than the soils of control sites. Moreover, analyzing the average values for the entire sample, significant differences in soil content in TOC and TN between objects M and C were demonstrated.

### 3.3. Activity of Soil Enzymes in Tested Soils

**Figure 6:**
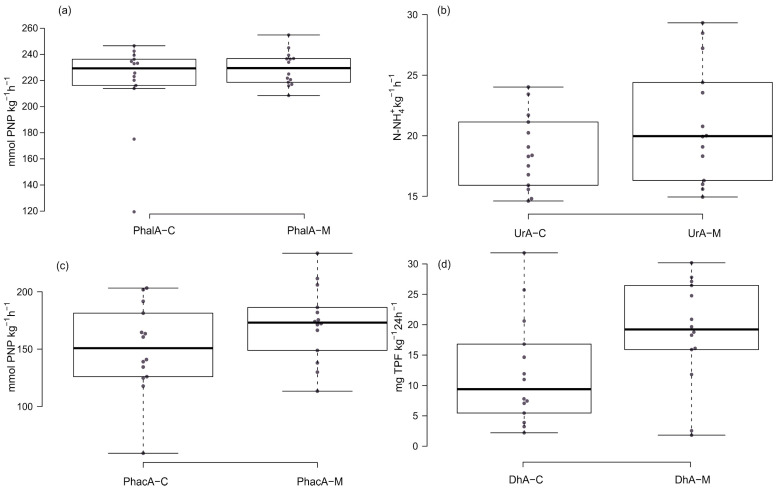
Box plots of soil enzyme activity: (**a**) activity of alkaline phosphatase (PhalA) (**b**) activity of urease (UrA), (**c**) activity of acid phosphatase (PhacA), and (**d**) activity of dehydrogenases (DhA). Explanations: M—sites occupied by *Cheilotoma musciformis*; C—control (unoccupied) sites. Solid centre line—the medians; box limits—the 25th and 75th percentiles; whiskers—1.5 times the interquartile range from the 25th and 75th percentiles, dots—outliers.

The activity of intracellular dehydrogenases (DhA) in surface soils from *Ch. musciformis* (M) was within wide limits of 1.28 to 30.19 mg TPF kg^−1^ 24 h^−1^ (mean 18.72 mg TPF kg^−1^ 24 h^−1^), and in the soils of objects C, from 2.21 to 31.83 mg TPF kg^−1^ 24 h^−1^ (12.12 mg TPF kg^−1^ 24 h^−1^) (Figure 6 and Table 2).

Urease activity (UrA) in the soils of sites with *Ch. musciformis* (M) ranged from 14.94 to 29.32 mg N-NH_4_^+^ kg^−1^ h^−1^ (mean 20.99 mg N-NH_4_^+^ kg^−1^ h^−1^), and in the soils of objects C from 14.61 to 24.02 mg N-NH_4_^+^ kg^−1^ h^−1^ (mean 18.67 mg N-NH_4_^+^ kg^−1^ h^−1^) (Figure 3 and Table 2).

The activities of acid phosphatase (PhacA) and alkaline phosphatase (PhalA) in the soils of site M ranged, respectively, from 113.44 to 233.68 mmol PNP kg^−1^ h^−1^ (average 172.12 mmol PNP kg^−1^ h^−1^ for PhacA) and from 208.48 to 254.88 mmol PNP kg^−1^ h^−1^ (average 229.34 mmol PNP kg^−1^ h^−1^ for PhalA) (Figure 6, Table 2). However, the activity of the tested phosphatases in control soils (C) ranged from 59.15 to 203.28 mmol PNP kg^−1^ h^−1^ (average 150.68 mmol PNP kg^−1^ h^−1^ for PhacA) and from 119.47 to 246.63 mmol PNP kg^−1^ h^−1^ (average 218.53 mmol PNP kg^−1^ h^−1^ for PhalA), (Figure 6, Table 2). The soils of most of the sites with *Ch. musciformis* (M) were characterized by statistically significantly higher activity of the enzymes tested (DhA, UrA, PhacA, and PhalA) compared to the enzyme activity of the soils of the control sites (C). Only in a few research areas was an inverse relationship found. Furthermore, by analyzing the average values of the entire sample, significant differences in the activity of the enzymes tested in the soil between objects M and C were demonstrated (Figure 6 and Table 2).

**Figure 7:**
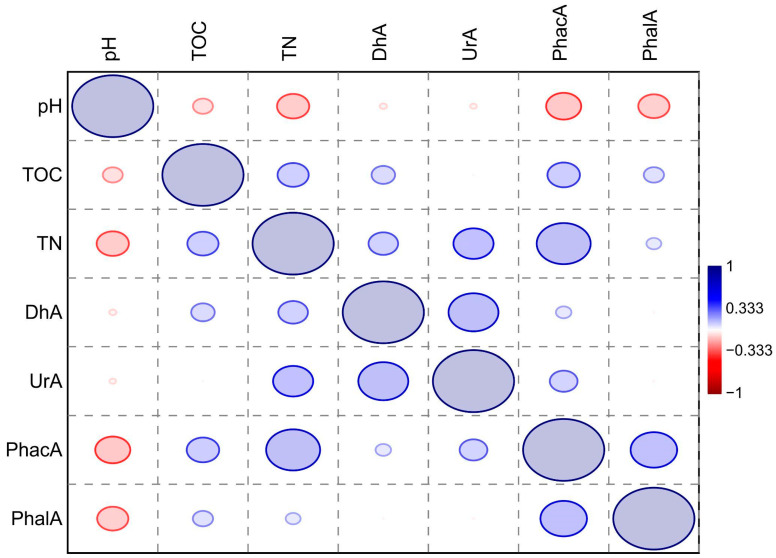
Significant correlation coefficients between enzyme activity and soil properties. Explanations: pH—acidity in 1 moldm^−3^ KCl; TOC—total organic carbon, TN—total nitrogen; DhA—activity of dehydrogenases; UrA—activity of urease; PhacA—activity of acid phosphatase; PhalA—activity of alkaline phosphatase. Colours and ellipses represent strength and direction of the correlation according to the legend.

Based on the simple correlation analysis (r), pH_KCl_ was shown to be significantly and negatively correlated with TN, PhacA and PhalA (respectively: r = −0.39; r = −0.38; r = −0.49 and r = −0.54). The total organic carbon (TOC) content was significantly and positively correlated with TN, PhacA, and PhalA (respectively: r = 0.35; r = 0.39 and r = 0.43). DhA, UrA, and both phosphatases were significantly and positively correlated with the total nitrogen (TN) content (respectively: r = 0.42; r = 0.45; r = 0.68 and r = 0.43). Moreover, DhA is positively correlated with UrA (r = 0.57) and PhacA with PhalA (r = 0.52), (Table 1 and Figure 7).

**Figure 8:**
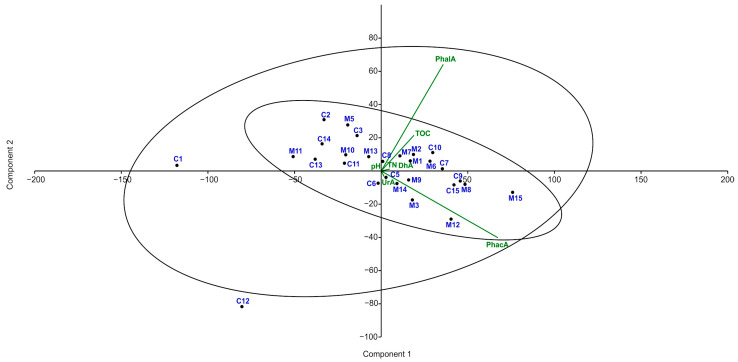
Principal component analysis (PCA) of the biochemical variables examined in the soil and the presence of the leaf beetle. M—sites occupied by *Cheilotoma musciformis*; C—control (unoccupied) sites. DhA—activity of dehydrogenases, UrA—activity of urease, PhacA—activity of acid phosphatase, PhalA—activity of alkaline phosphatase, TOC—content of organic carbon, TN—total nitrogen, pH—pH in 1 moldm^−3^ KCl. The blue color letters represent the sites occupied by Cheilotoma musciformis and control (unoccupied) sites. The green color letters represent the Principal component of the biochemical variables examined in the soil. The circle area represents the relevance of the presence of leaf beetles.

According to PCA (PC1 = 72%, PC2 = 18%), there was no clear distinction between the examined sites according to their chemical properties, except two outliers (K1 and K12) (Figure 8). For two pairs of most correlated variables (DhA with UrA, and TN with PhacA), principal components (1) were extracted and used in further analyses.

Among a set of GLMs, two models have the lowest values of AIC and only these models were significant (Table 3). These two models included only two variables: TOC and PC1 of DhA with UrA (being highly correlated).

## 4. Discussion

In recent decades, there has been an increase in interest in research on the phenomenon known as feedback between plants and the soil and the processes occurring in it [51,52] and plants and insects [53,54,55,56]. There is also research on the plant–insect–microbe interaction [57]. However, there is a lack of information on the relationship between the soil and terrestrial insects. In this work, the authors attempted to describe the complex interactions that can occur between the soil environment and terrestrial organisms.

### 4.1. The Activity of Enzymes in Soil

Enzyme activity in the soil environment is considered one of the most sensitive indicators of habitat functioning and soil fertility [58]. In these studies, there was a trend indicating significantly higher enzymatic activity in soils of sites with *Ch. musciformis* (M) compared to soils of sites without the beetle. The sites (M), were subject to extensive grazing of farm animals, or the grasslands inhabited by the beetle were recently exposed (cleared of bushes). However, the control sites have not been used for a long time, and some of the most overgrown areas have recently been intensively grazed after the shrubs were removed as part of active protection of xerothermic grasslands. Some of the control grasslands were inhabited by this taxon in the past but were abandoned by it as a result of too intense grazing, which resulted in the disappearance of the host plant before the beetle’s reproductive period (May). Research by Futa et al. [35,36] and Bielińska et al. [42] conducted, among others, on xerothermic grasslands in Eastern Poland also indicate higher enzymatic activity in the soils of sites with extensive grazing of farm animals compared to sites without grazing. On the other hand, some studies indicate that too-intensive grazing has a negative impact on vegetation and epigeic entomofauna of the steppes [59]. Futa et al. [35,36] and Bielińska et al. [42] showed that extensive grazing has a beneficial effect on habitat biodiversity and the fertility and health of soil [35,36,42]. Soil properties determined by their chemical composition (including TOC and TN content) and enzymatic activity are considered a reliable indicator of habitat fertility [60,61]. This thesis is confirmed by our research. The correlation analysis performed showed a significant correlation coefficient between the activity of all enzymes tested and the amount of TN in the soils and between TN, PhacA, and PhalA and the TOC content in the soils. Additionally, among the GLM set, only two models included two highly correlated variables: TOC and PC1 of DhA with UrA. Santo-Silva et al. [62] showed that soil fertility is an important habitat-forming factor that influences the abundance of gall-inducing insects in the formation of caatinga plants in Brazil.

### 4.2. The Physicochemical Properties of Soils

The condition of the habitat and the activity of microorganisms and the enzymes they secrete are determined by the availability of nutrients [63]. The physicochemical and biochemical properties of soil are also significantly influenced by chemical compounds released from plant roots in the form of dead cells and other debris or in the form of root “exudates” [64]. Root exudates are organic chemicals released into the soil by a living and intact root system [65]. These products have different chemical compositions depending on the species, type, and growth phase of the plant. Plants producing exudates of a specific composition stimulate specific groups of microorganisms and soil enzymes that catalyze the mineralization of organic matter and release C, N, S, and P compounds available to plants [66]. On the contrary, soil microorganisms are capable of changing the chemical composition of root exudates and thus plant physiology, enabling host plants to colonize nutrient-poor soils [65,67]. Root exudation activates microorganisms to secrete certain compounds, e.g., acidic components, hydroxyl ions, phenols, phosphatase, etc., which stimulates plant growth and resistance to stress factors [68].

The obtained values of soil enzyme activity reflect the in situ state determined not only by current conditions in the soil, but also by the history of events preceding the determination, including climatic conditions, applied treatments, soil location and relief, and the presence of plants. Each type of soil, depending on its origin and development conditions, is different in terms of organic matter content and microbial activity and is also characterized by its own level of enzymatic activity. Therefore, there are no limit numbers that determine the activity of enzymes under given soil conditions. A reliable assessment of soil quality is provided by testing a number of enzymes, which allows for recording changes in the soil environment [37,69,70].

### 4.3. Soil Condition Consequences for the Occurrence of the Beetle

The higher enzymatic activity of soils in *Ch. musciformis* sites than in the control sites can indicate the dependence of the occurrence of this beetle on the presence of vegetation at a certain development stage. These are, for example, loose patches of xerothermic grasslands, where extensive grazing is carried out, which affects the behavior of the preferred host plant species. In Poland, this beetle prefers clumps of legumes (mainly *Onobrychis*) growing in habitats with exposed fragments of the parent rock (gypsum or chalk). This is probably the result of selectivity in terms of egg-laying sites. Exposed soil fragments can also maintain a specific ground microclimate that affects the development of larvae. *Cheilotoma* is a thermophilic species, so warming the soil can accelerate its development in spring. This can be important in locations in the northern range of the species. For this reason, when planning the active protection of xerothermic grasslands, which are the habitat of the beetle, it is necessary to take into account the structure of the vegetation and possible changes in the biochemical properties of the soil. Both the lack of treatments, such as bush removal and extensive grazing, and too intense grazing (leading on the one hand to the elimination of vegetation during the grazing period, and on the other hand to overfertilization of the soil and the spread of nitrophilous species), have an adverse effect on the occurrence of *Ch. musciformis*.

Conscious disruption of any edaphic factor or uncontrolled transformation of vegetation in these habitats can quickly deprive this species of the possibility of existence in the only small area of its occurrence in Poland. This is the first work to attempt to find the causes of the extinction of this taxon in Poland in relation to the enzymatic activity of soils in the sites studied with and without the beetle.

## 5. Conclusions

Soils in beetle-inhabited sites exhibited significantly higher levels of enzyme activity, total organic carbon, and total nitrogen, along with lower pH_KCl_ compared to control sites. Higher enzymatic activity of soils in sites with the beetle than in the control sites may indicate the dependence of the occurrence of this beetle on the presence of patches of xerothermic grasslands extensively grazed. Given that grazing practices influence the behavior of preferred host plant species, effective protection planning for *Ch. musciformis*-inhabited grasslands, should carefully consider changes in soil biochemical properties and vegetation structure.

## Figures and Tables

**Table 1:** Mean values and standard deviation (±) of chemical properties of the tested soils (N = 28).

Site	pH_KCl_		TOC		TN	
	M	C	M	C	M	C
1	6.84 ± 0.01 ^a^	7.27 ± 0.03 ^b^	54.11 ± 2.54 ^a^	15.08 ± 0.24 ^b^	3.92 ± 0.03 ^a^	7.27 ± 0.03 ^b^
2	6.87 ± 0.03 ^a^	7.06 ± 0.00 ^b^	44.31 ± 0.86 ^a^	51.46 ± 1.53 ^b^	2.52 ± 0.02 ^a^	1.40 ± 0.03 ^b^
3	6.97 ± 0.01 ^a^	7.06 ± 0.00 ^b^	46.71 ± 1.03 ^a^	51.91 ± 0.55 ^b^	4.06 ± 0.03 ^a^	1.40 ± 0.06 ^b^
5	6.86 ± 0.02 ^a^	6.91 ± 0.01 ^b^	59.83 ± 0.65 ^a^	62.06 ± 2.28 ^a^	1.68 ± 0.03 ^a^	2.94 ± 0.04 ^b^
6	6.88 ± 0.01 ^a^	7.01 ± 0.01 ^b^	61.14 ± 1.70 ^a^	54.05 ± 1.58 ^b^	1.40 ± 0.01 ^a^	2.52 ± 0.02 ^b^
7	6.97 ± 0.00 ^a^	6.90 ± 0.02 ^b^	51.93 ± 1.72 ^a^	53.65 ± 2.83 ^a^	1.96 ± 0.02 ^a^	2.24 ± 0.03 ^b^
8	7.01 ± 0.01 ^a^	6.98 ± 0.01 ^b^	62.45 ± 1.45 ^a^	40.14 ± 0.50 ^b^	3.50 ± 0.01 ^a^	1.54 ± 0.04 ^b^
9	6.79 ± 0.02 ^a^	7.02 ± 0.00 ^b^	73.78 ± 0.40 ^a^	57.53 ± 0.46 ^b^	3.36 ± 0.02 ^a^	4.48 ± 0.02 ^b^
10	6.90 ± 0.01 ^a^	6.75 ± 0.02 ^b^	49.91 ± 0.36 ^a^	57.59 ± 0.76 ^b^	3.78 ± 0.03 ^a^	2.24 ± 0.02 ^b^
11	6.84 ± 0.00 ^a^	7.07 ± 0.02 ^b^	34.79 ± 1.51 ^a^	28.70 ± 0.39 ^b^	2.10 ± 0.02 ^a^	1.96 ± 0.05 ^a^
12	6.86 ± 0.00 ^a^	7.25 ± 0.03 ^b^	33.2 ± 0.14 ^a^	9.84 ± 0.16 ^b^	2.80 ± 0.03 ^a^	0.70 ± 0.00 ^b^
13	7.27 ± 0.02 ^a^	6.97 ± 0.02 ^b^	62.51 ± 0.01 ^a^	18.57 ± 0.07 ^b^	2.52 ± 0.00 ^a^	2.10 ± 0.04 ^b^
14	7.27 ± 0.02 ^a^	7.25 ± 0.01 ^a^	61.63 ± 1.73 ^a^	33.24 ± 0.33 ^b^	3.64 ± 0.01 ^a^	1.96 ± 0.01 ^b^
15	6.85 ± 0.01 ^a^	6.82 ± 0.00 ^b^	43.00 ± 0.20 ^a^	55.86 ± 2.32 ^b^	4.90 ± 0.02 ^a^	5.04 ± 0.05 ^a^

Explanations: M—site of *Cheilotoma musciformis*; C—control area; pH_KCl_—pH in 1 moldm^−3^ KCl; TOC—total organic carbon, TN—total nitrogen; ^a,b^—different small letters indicate significant difference at *p* ≤ 0.05 for land use.

**Table 2:** Mean values and standard deviation (±) of enzymatic activity of the tested soils (N = 28).

Site	DhA		UrA		PhacA		PhalA	
	M	C	M	C	M	C	M	C
1	15.90 ± 0.48 ^a^	7.07 ± 0.33 ^b^	28.47 ± 0.33 ^a^	21.70 ± 0.12 ^b^	172.27 ± 0.82 ^a^	59.15 ± 2.54 ^b^	236.41 ± 0.41 ^a^	175.13 ± 0.74 ^b^
2	1.82 ± 0.40 ^a^	2.21 ± 0.07 ^a^	14.94 ± 0.11 ^a^	14.80 ± 0.64 ^a^	173.96 ± 3.42 ^a^	117.85 ± 3.96 ^b^	245.04 ± 2.22 ^a^	233.22 ± 0.41 ^b^
3	2.55 ± 0.45 ^a^	3.90 ± 0.41 ^b^	19.08 ± 0.24 ^a^	14.61 ± 0.35 ^b^	186.41 ± 3.08 ^a^	139.18 ± 2.35 ^b^	218.64 ± 1.10 ^a^	234.62 ± 3.30 ^b^
5	16.10 ± 0.59 ^a^	16.80 ± 0.47 ^a^	16.30 ± 0.17 ^a^	18.38 ± 0.60 ^b^	130.04 ± 1.27 ^a^	164.58 ± 2.12 ^b^	233.99 ± 1.70 ^a^	216.23 ± 0.22 ^b^
6	19.66 ± 1.69 ^a^	7.81 ± 0.60 ^b^	19.93 ± 0.36 ^a^	15.91 ± 0.35 ^b^	181.98 ± 3.03 ^a^	163.47 ± 0.90 ^b^	239.39 ± 3.84 ^a^	213.92 ± 0.39 ^b^
7	11.84 ± 0.49 ^a^	7.46 ± 0.34 ^b^	15.59 ± 0.04 ^a^	18.29 ± 0.16 ^b^	166.43 ± 1.28 ^a^	191.71 ± 0.63 ^b^	236.61 ± 0.68 ^a^	242.53 ± 0.30 ^b^
8	26.46 ± 2.96 ^a^	5.47 ± 0.29 ^b^	20.00 ± 0.17 ^a^	16.78 ± 0.38 ^b^	206.07 ± 0.95 ^a^	160.55 ± 1.81 ^b^	236.85 ± 0.86 ^a^	232.93 ± 5.80 ^a^
9	24.77 ± 0.39 ^a^	20.61 ± 0.09 ^b^	18.31 ± 0.43 ^a^	21.13 ± 0.17 ^b^	175.49 ± 8.11 ^a^	203.28 ± 9.19 ^b^	217.10 ± 0.71 ^a^	239.37 ± 0.45 ^b^
10	18.77 ± 2.36 ^a^	14.66 ± 0.20 ^b^	23.55 ± 0.43 ^a^	19.07 ± 0.10 ^b^	138.15 ± 0.38 ^a^	181.38 ± 0.39 ^b^	220.67 ± 0.53 ^a^	246.63 ± 1.04 ^b^
11	20.88 ± 2.19 ^a^	31.83 ± 0.47 ^b^	15.99 ± 0.51 ^a^	23.43 ± 0.49 ^b^	113.44 ± 0.79 ^a^	141.04 ± 1.06 ^b^	208.48 ± 1.43 ^a^	223.09 ± 3.68 ^b^
12	18.27 ± 0.39 ^a^	3.23 ± 0.24 ^b^	29.32 ± 0.06 ^a^	17.51 ± 0.11 ^b^	211.58 ± 1.64 ^a^	134.50 ± 1.48 ^b^	224.98 ± 0.48 ^a^	119.47 ± 0.40 ^b^
13	27.12 ± 0.26 ^a^	25.72 ± 0.35 ^b^	27.22 ± 0.22 ^a^	24.02 ± 0.79 ^b^	148.85 ± 2.26 ^a^	126.08 ± 1.34 ^b^	221.79 ± 0.92 ^a^	220.22 ± 0.62 ^a^
14	27.80 ± 0.27 ^a^	11.94 ± 0.21 ^b^	20.77 ± 0.04 ^a^	15.57 ± 0.55 ^b^	171.34 ± 1.16 ^a^	124.91 ± 1.11 ^b^	215.96 ± 1.53 ^a^	225.74 ± 0.96 ^b^
15	30.19 ± 0.06 ^a^	11.00 ± 0.07 ^b^	24.41 ± 0.02 ^a^	20.24 ± 0.62 ^b^	233.68 ± 2.84 ^a^	201.86 ± 0.44 ^b^	254.89 ± 0.71 ^a^	236.30 ± 1.23 ^b^

Explanations: M—site of *Cheilotoma musciformis*; C—control area; DhA—activity of dehydrogenases; UrA—activity of urease; PhacA—activity of acid phosphatase; PhalA—activity of alkaline phosphatase; ^a,b^—different small letters indicate significant difference at *p* ≤ 0.05 for land use.

**Table 3:** Set of generalized linear models built on variables of soil chemistry in order to explain presence of *Chelitoma musciformis*.

Model	Variables				d.f.	AIC	Chi2	*p*
1	PC−1	TOC	–	–	2	37.9	6.9	0.032
2	PC−1	–	–	–	1	38.8	4.0	0.046
3	TOC	–	–	–	1	39.2	3.6	0.058
4	PC−2	PC−1	–	–	2	39.3	5.5	0.065
5	PC−2	PC−1	TOC	–	3	39.7	7.1	0.068
6	PC−1	TOC	PhalA	–	3	39.9	6.9	0.075
7	PC−1	PhalA	–	–	2	39.9	4.9	0.087
8	PC−2	–	–	–	1	40.2	2.6	0.107
9	PC−2	TOC	–	–	2	40.5	4.3	0.114
10	TOC	PhalA	–	–	2	41.2	3.6	0.162
11	PC−2	PC−1	PhalA	–	3	41.2	5.6	0.133
12	PhalA	–	–	–	1	41.4	1.4	0.230
13	PC−2	PC−1	TOC	PhalA	4	41.7	7.1	0.129
14	PC−2	PhalA	–	–	2	42.0	2.8	0.250
15	PC−2	TOC	PhalA	–	3	42.5	4.4	0.226
	Intercept	–	–	–	–	40.8	–	–

Explanations: d.f.—degree of freedom, AIC—Akaike Information Criterion, *p*—*p*-value, PC-1 = DhA with UrA, PC-2 = TN with PhacA.

## Data Availability

The data presented in this study are available on request from the first author.

## Supplementary Materials

The following supporting information can be downloaded at: https://www.mdpi.com/article/10.3390/insects15050307/s1, KMZ file (Cheilotoma_sites.kmz).
